# Zi Shen Huo Luo Formula Enhances the Therapeutic Effects of Angiotensin-Converting Enzyme Inhibitors on Hypertensive Left Ventricular Hypertrophy by Interfering With Aldosterone Breakthrough and Affecting Caveolin-1/Mineralocorticoid Receptor Colocalization and Downstream Extracellular Signal-Regulated Kinase Signaling

**DOI:** 10.3389/fphar.2020.00383

**Published:** 2020-04-03

**Authors:** Xiaotong Song, Yue Zhao, Shijun Wang, Yuan Wang, Qian Chen, Haijun Zhao, Hua Wang, Sheng Tian, Huayun Yu, Zhichun Wu

**Affiliations:** ^1^College of Traditional Chinese Medicine, Shandong University of Traditional Chinese Medicine, Jinan, China; ^2^Shandong Co-innovation Center of Classic TCM Formula, Shandong Provincial Education Department, Jinan, China; ^3^Department of Geriatric Medicine, Hospital Affiliated to Shandong University of Traditional Chinese Medicine, Jinan, China

**Keywords:** hypertension, left ventricular hypertrophy, aldosterone breakthrough, Zi Shen Huo Luo formula, mineralocorticoid receptor, EGFR signaling pathway

## Abstract

Left ventricular hypertrophy (LVH) is an important characteristic of hypertensive heart disease. Renin-angiotensin system (RAS) blockers have been shown to be effective drugs for the reversal of LVH. Clinical and experimental studies have shown that Zi Shen Huo Luo Formula (ZSHLF) can improve the efficacy of perindopril in the treatment of hypertensive LVH, but its mechanism is unclear. This study aimed to investigate the possible mechanism to improve the efficacy of perindopril. First, we identified 23 compounds in ZSHLF by ultra performance liquid chromatography/tandem mass spectrometry (UPLC-MS/MS) analysis, among which ferulic acid, caffeic acid, vanillic acid, berberine, rutin, quercetin, kaempferol, stachydrine, and tiliroside have been reported to reduce blood pressure and exhibit cardioprotective effects. Second, we treated spontaneously hypertensive rats (SHRs) with perindopril and ZSHLF for 12 continuous weeks and found that chronic use of perindopril could increase the aldosterone (ALD) levels and cause aldosterone breakthrough (ABT). ZSHLF combined with perindopril reduced the ALD levels, interfered with ABT, decreased blood pressure, improved left ventricular diastolic dysfunction, and decreased the collagen volume fraction; these effects were superior to those of perindopril alone. In vitro experiments, ALD-induced cardiomyocytes (H9c2 cells) and cardiac fibroblasts were treated with ZSHLF-containing serum, which suppressed ALD-induced cardiomyocyte hypertrophy and cardiac fibroblast proliferation, increased mineralocorticoid receptor (MR) and Cav-1 colocalization and decreased phosphorylated epidermal growth factor receptor (pEGFR) and phosphorylated extracellular signal-regulated kinase (pERK) protein expression the cells. In conclusion, ZSHLF can interfere with ABT and affect the pathological role of ALD by affecting MR and Cav-1 interactions and EGFR/ERK signaling pathway. These effects represent a possible mechanism by which ZSHLF improves the efficacy of angiotensin-converting enzyme inhibitors (ACEIs) in hypertensive LVH treatment. However, the major bioactive components or metabolites responsible for the effects and the implications of these findings in patients need further verification.

## Introduction

Left ventricular hypertrophy (LVH) is an important characteristic of hypertensive heart disease that mainly involves cardiomyocyte hypertrophy and fibrosis due to cardiac fibroblast proliferation. LVH is the inciting event of several adverse cardiovascular events, such as arrhythmia, heart failure, myocardial infarction, and even sudden death (Jekell et al., 2018; Slivnick and Lampert, 2019). Reversing hypertensive LVH and reducing the occurrence of cardiovascular complications and mortality are the focuses of clinical hypertension treatments (Xiong et al., 2013). Renin-angiotensin system (RAS) blockers, such as angiotensin-converting enzyme inhibitors (ACEIs) and angiotensin receptor blockers (ARBs), are applied clinically to manage pathological cardiac hypertrophy (Hellawell and Margulies, 2012). RAS blockers lower plasma aldosterone (ALD) levels at the beginning of treatment, but the ALD levels may return to or exceed the pretreatment values as the treatment time increases. This phenomenon is known as aldosterone breakthrough (ABT), and it may limit the beneficial effects of RAS blockers (Christ et al., 2004; Ubaid-Girioli et al., 2007). Furthermore, high ALD levels can cause pathological remodeling in the context of heart disease, fibrosis, and LVH (Takeda et al., 2000; Wynn, 2008; Chou et al., 2018), participating in the pathological progression of cardiovascular clinical events. ALD receptor antagonists, such as spironolactone and eplerenone, are commonly used in clinical practice. However, chronic use may cause adverse reactions (Toyonaga et al., 2011), limiting their application. Thus, strongly interfering with ABT and controlling the cardiac hypertrophy induced by elevated ALD are important strategies for improving the efficacy of RAS blockers and reducing the risk of cardiovascular risk events.

ALD-mediated fibrosis and detrimental cardiac remodeling are associated with mineralocorticoid receptor (MR) activation in the cardiovascular system; these events are mainly mediated by steroid receptors localized at the plasma membrane instead of by classic nuclear hormone receptors (Williams, 2013). There is evidence that the activation of ALD described above may involve interaction with caveolin-1 (Cav-1), which binds to MR and localizes to the cell membranes of cardiac cells, where it mediates intracellular events *via* cell surface receptors (Baudrand et al., 2014). Upon excessive ALD stimulation, MR is released from the membrane (Krug et al., 2011), after which it transactivates epidermal growth factor receptor (EGFR), activates MAPK and ERK1/2 (Ricchiuti et al., 2011; Dooley et al., 2012; Chen et al., 2013), and enters the nucleus to regulate the transcription of downstream signaling molecules and to promote cardiomyocyte hypertrophy and myocardial fibrosis (Navaneethan and Bravo, 2013).

Zi Shen Huo Luo Formula (ZSHLF), a traditional Chinese medicine (TCM) herbal formula, is composed of six crude drug materials. Previous studies have confirmed that ZSHLF combined with perindopril can improve the diastolic function of the left ventricle in spontaneously hypertensive rats (SHRs) and patients with hypertensive LVH (Wang and Gao, 2013; Yang et al., 2018). These results are consistent with the results of modern pharmacological studies reported in the literature, which indicate that herbal medicines in ZSHLF such as *Scrophularia ningpoensis* Hemsl., *Achyranthes bidentata* Bl., *Coptis chinensis* Franch., *Paeonia suffruticosa* Andr., *Leonurus japonicu*s Houtt., and *Cinnamomum cassia* Presl. can lower blood pressure, inhibit cardiomyocyte hypertrophy and ventricular remodeling and have protective effects on the heart (Mhatre et al., 2009; Huang et al., 2012; Wojtyniak et al., 2013; Dan et al., 2016; Sobhani et al., 2017).

Although its effects are known, the chemical composition and pharmacological mechanism of ZSHLF are poorly understood. The present study aimed to identify the effects of ZSHLF treatment on ABT, the interaction between MR and Cav-1 in myocardial tissue and downstream EGFR signaling.

## Materials and Methods

### Animals

Male 15-week-old SHRs (*n*=24, weighing 260–300 g), male 15-week-old Wistar-Kyoto (WKY) rats (*n*=8, weighing 280–320 g), and male and female 6-week-old Sprague-Dawley (SD) rats (*n*=20, half male and half female, weighing 180–220 g) were provided by Vital River Laboratory Animal Technology Co., Ltd. (Beijing, China) and were maintained at the Animal Experimental Center of Shandong University of Traditional Chinese Medicine (Shandong, China). All rats were maintained in an environment with a controlled temperature (23–24 °C) and relative humidity (50–60%) and had free access to water and food. All experiments were conducted with the approval of the Institutional Animal Care and Use Committee and were in compliance with the Use and Care of Laboratory Animals guidelines.

### Drug Materials

ZSHLF consists of six Chinese herbal medicines, including the dried roots of *Scrophularia ningpoensis* Hemsl. and *Achyranthes bidentata* Bl., the dried rhizome of *Coptis chinensis* Franch., the dried root cortex of *Paeonia suffruticosa* Andr., the dried aerial part of *Leonurus japonicu*s Houtt., and the dried bark of *Cinnamomum cassia* Presl., at a fixed ratio of 20:15:12:12:20:3, respectively. These herbs were purchased from the Affiliated Hospital of Shandong University of Traditional Chinese Medicine (Shandong, China) and identified by Prof. H.Y. Liu. The details of the drug materials are given in **Supplementary Table 1**. The above drugs were macerated in eight volumes of distilled water for 60 min and decocted for 30 min. After filtration, the remaining drugs were added to six volumes of water and decocted for 20 min. The filtrates were mixed, concentrated to 8.2 g crude drug/ml and filtered through a 0.2 μm membrane. Perindopril (Servier Tianjin Pharmaceutical Co., Ltd., Tianjin, China) was dissolved in distilled water to make a suspension of 0.1 mg/ml before use and stored at 4 °C.

### Preparation of ZSHLF-Containing Serum

Three days after adaptive breeding, the SD rats were randomly divided into blank control serum group and ZSHLF-containing serum group. The rats in the ZSHLF-containing serum group received ZSHLF intragastrically at a dose of 8.2 g/kg twice daily for 5 d. The rats in the blank control serum group were given equal volumes of normal saline (NS). The rats were starved for 12 h after the last administration of ZSHLF at a 1-day dosage. One hour after the last dose, blood was collected; the serum was separated, inactivated at 56 °C and filtered with a 0.22 μm membrane. The serum was frozen at −80 °C.

### Chemical Composition Analysis of ZSHLF by UPLC-MS/MS

Ultra performance liquid chromatography/tandem mass spectrometry (UPLC-MS/MS) analysis was performed on a Thermo Fisher UltiMate 3000 RS [Thermo Fisher Scientific (China) Co., Ltd., China] system coupled to a Q-Exactive high-resolution mass spectrometer [Thermo Fisher Scientific (China) Co., Ltd., China]. Two microliters of sample solution was injected into a Thermo Hypersil GOLD column (100×2.1 mm, 1.9 μm). After optimizing the chromatographic conditions, the column temperature was adjusted to 35 °C, and the autosampler was conditioned at 10 °C. A 200 μl sample solution was injected into the system for analysis. The mobile phase consisted of 0.1% formic acid aqueous solution (A) and acetonitrile (B). A linear gradient elution was applied (0–5 min, 2–20% B; 5–10 min, 20–50% B; 10–15 min, 50–80% B; 15–25 min, 80–95% B; 25–30 min, 95–2% B) at a flow rate of 0.3 ml/min. Mass spectrometric detection was carried out with an electrospray ionization (ESI) interface set in positive or negative ionization mode.

### Rat Grouping and Drug Administration

After 1 week of acclimation, 24 SHRs were randomly divided into three groups (*n*=8/group): SHR control group (SHR group), perindopril group (PEP group), combination of ZSHLF, and perindopril group (ZSHLF group). Eight WKY rats of the same age were used as the normal control group (WKY group). The ZSHLF group received 1 mg/kg/d perindopril in the morning and received 8.2 g/kg/d ZSHLF in the afternoon. The PEP group received the same dosage of perindopril in the morning and the same amount of NS in the afternoon. The SHR and WKY groups were orally fed the same volumes of NS twice daily. The rats were treated for 12 weeks.

### Blood Pressure Measurement

After 0, 4, 8, and 12 weeks of treatment, the tail-cuff method was used to measure systolic blood pressure (SBP) and diastolic blood pressure (DBP) with a BP-2010A intelligent noninvasive blood pressure meter (Softron Beijing Biotechnology Co., Ltd., China). Each rat was placed in a heated tube (38 °C) for 10–15 min before measurement to raise its body temperature. Each group of rats was measured in parallel, and each rat was measured three times. The mean of the three readings was regarded as the value of that parameter for the animal.

### Blood Collection and Serum ALD Detection

At weeks 0, 4, 8, and 12, rats were anesthetized with 4% isoflurane and maintained on 2% isoflurane, and blood samples were collected from the jugular vein. The blood samples were centrifuged at 3,500 r/min for 10 min to isolate serum. The serum ALD levels were measured with an enzyme-linked immunosorbent assay (ELISA).

### Hemodynamic Recording and Tissue Specimens

After 12 weeks of treatment, rats were anesthetized by intraperitoneal injection of 7% chloral hydrate (3 ml/kg). A catheter connected to a pressure sensor was inserted into the right carotid artery and advanced into the left ventricle to measure hemodynamic parameters. After 5 min of stabilization, the left ventricular systolic pressure (LVSP), left ventricular end diastolic pressure (LVDP), and changes in the maximal rate of the pressure increase in the contract phase (+dp/dtmax) and the maximal rate of the pressure decrease in the diastole phase (−dp/dtmax) in the left ventricle were observed and recorded with LabChart software. After hemodynamic recording, the myocardial tissues were cut from the apex and immediately washed with ice-cold phosphate buffer. The tissue samples were cut into two segments: one segment was immediately frozen in liquid nitrogen and stored at −80 °C until analysis, and the other segment was fixed in 4% paraformaldehyde (PFA) for Masson staining.

### Masson Staining

The left ventricular apex tissues of rats were fixed with 4% PFA for 24 h, embedded in paraffin, and serially sliced to a thickness of 5 μm. The sections were stained with modified Masson’s trichrome staining solution and observed under a light microscope. Image-Pro Plus 6.0 image analysis software was used to analyze and calculate the collagen volume fraction (CVF) with the following equation: CVF = (collagen area/total cardiac muscle area) ×100%.

### Cell Culture and Treatment

The H9c2(2-1) cell line was purchased from the Cell Bank of Type Culture Collection of the Chinese Academy of Sciences (Shanghai, China), and the rat cardiac fibroblast (RCF) cell line was purchased from Procell Life Science & Technology Co., Ltd. (Wuhan, China). The H9c2 cells and RCFs were grown in DMEM (HyClone, USA) supplemented with 10% fetal bovine serum (FBS) and 1% penicillin/streptomycin in a humidified atmosphere of 5% CO_2_ at 37°C. The cells were divided into the following groups: blank serum control group (BC group), model (ALD group), ALD+ZSHLF (ZSHLF group). The cells in the ZSHLF group were treated with various concentrations of ZSHLF-containing serum (5%, 10%, and 20%) for 2 h, while the cells in the BC group and ALD group were treated with 10% blank serum for 2 h. The cells in the ALD group and ZSHLF group were incubated with ALD for 30 min.

### Cardiac Fibroblast Proliferation Assay

Cardiac fibroblast proliferation was analyzed using the Cell Counting Kit-8 (CCK-8) assay (CP002, Signalway Antibody, USA) according to the instructions. After RCFs were treated by different methods, the cells were resuspended in CCK-8 reagent and serum-free essential minimal medium at a volume ratio of 1:10. The cells were incubated for 1 h at 37°C in a 5% CO_2_ incubator (ThermoForma3111). A microplate reader (MK3, Finnpipette) was used to measure the absorbance at 450 nm.

### α-Actinin Immunofluorescence Labeling for Measurement of the Cell Surface Area

The surface areas of cardiomyocytes were measured by α-actinin immunofluorescence labeling. After treatment, H9c2 cells were washed with PBS, fixed in 4% PFA for 20 min and then blocked with goat serum for 1 h at room temperature. The H9c2 cells were then incubated with an anti-α-actinin antibody (1:60, AB50591, Abcam, UK) overnight at 4 °C. The cells were washed with PBS and incubated with secondary antibodies conjugated to FITC (bs-0346R-FITC, Beijing Solarbio Science & Technology Co., Ltd., Beijing, China) for 1 h in a dark, moist chamber. After washing three times with PBS, the cells were stained with DAPI (04002, Beijing Solarbio Science & Technology Co., Ltd., Beijing, China) for 10 min and examined under a fluorescence microscope (Olympus BX51). Then, the cells were observed and photographed under a 400× fluorescence microscope; five different fields of view were randomly selected for each group. The cell surface areas were analyzed using ImageJ software.

### Immunofluorescence and Colocalization Analysis

After the cells were treated, fixed, and blocked with 10% goat serum, they were incubated overnight with an anti-MR antibody (1:60; rabbit polyclonal, AB64457, Abcam, UK) and an anti-Cav-1 antibody (1:60; mouse monoclonal, AB85491, Abcam, UK). The samples were then treated with FITC-conjugated anti-mouse IgG (1:500, ZF-0312-FITC, Zsbio, Beijing, China) and anti-rabbit IgG/RBITC (ZF-0316-RBITC, Zsbio, Beijing, China) secondary antibodies for 1 h at room temperature. The slides were observed and photographed under an Olympus IX73 fluorescence inverted microscope (IX73-DP80, Olympus Corporation, Tokyo, Japan). ImageJ software was used for colocalization analysis and indirect assessment of the sizes of the colocalized pixel areas with Pearson’s correlation coefficient (Pearson’s r) in each set of images.

### Western Blot Analysis

Western blot analysis was performed on protein extracts of tissues and cells. The proteins were first resolved by 5% and 10% SDS-PAGE and electrotransferred onto PVDF membranes (Millipore, USA). After blocking, the membranes were incubated with primary antibodies overnight at 4 °C. The following primary antibodies were used: anti-EGFR antibody (1:1,200, rabbit monoclonal antibody, AB52894, Abcam, UK), anti-EGFR (phosphor Y1068) antibody (1:800, rabbit polyclonal antibody, AB5644, Abcam, UK), anti-ERK1+ERK2 antibody (1:1000, rabbit polyclonal antibody, AB17942, Abcam, UK), anti-phosphor-Erk1/2 (Thr202/Tyr204) antibody (1:600, rabbit monoclonal, 4370S, Cell Signaling Technology, Inc., USA), and anti-β-actin antibody (1:1000, rabbit polyclonal antibody, AB8227, Abcam, UK). Then, the membranes were incubated with a secondary antibody (1:3000, goat anti-rabbit Ig G, AB205718, Abcam, UK) at room temperature for 1 h before being incubated with an electrochemiluminescence reagent for 30 s to 2 min and exposed to Kodak film. The data are presented as the relative optical density (ROD, relative to β-actin) and were determined using ImageJ software.

### Statistical Analysis

The Statistical Package for the Social Sciences (SPSS) 22.0 was used for statistical analysis. The data are expressed as the means ± standard deviations ($\bar{x}$ ± *s*). Statistical significance was determined by one-way ANOVA followed by Bonferroni’s multiple comparison test. A value of *P* < 0.05 was considered to indicate statistical significance.

## Results

### Identification of the Components of ZSHLF

**Figure 1:**
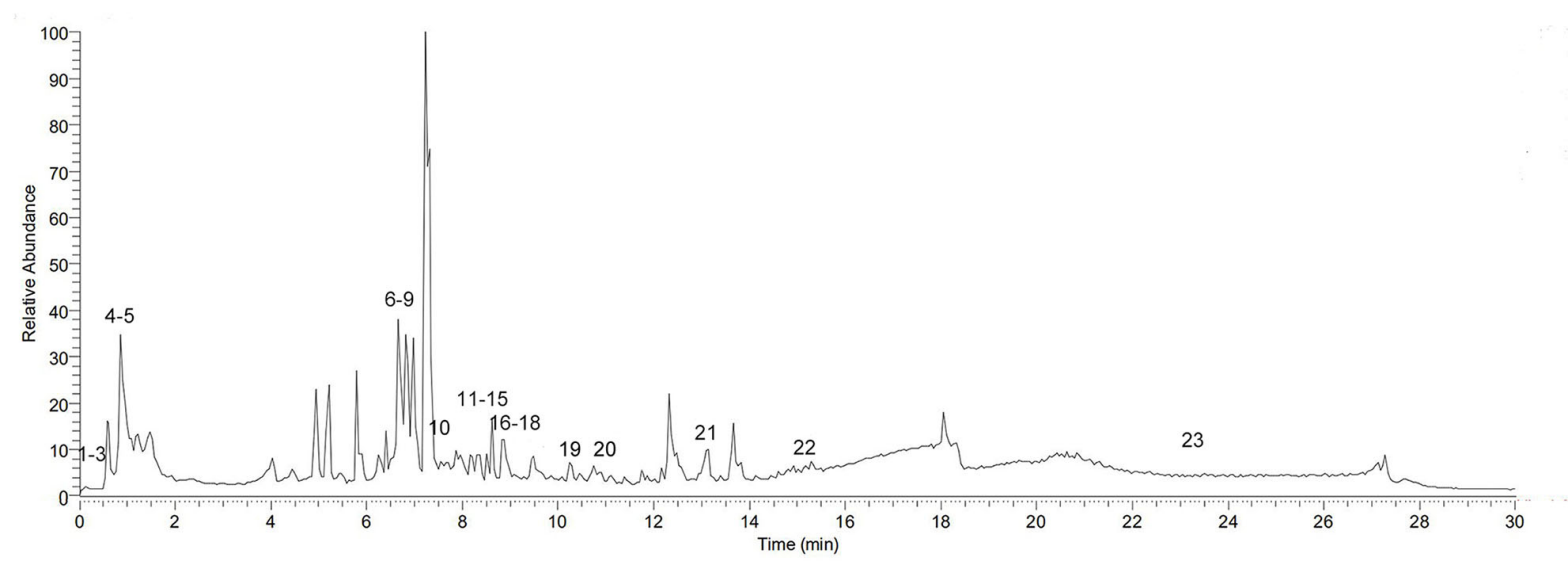
Base peak ion (BPI) chromatogram of Zi Shen Huo Luo Formula (ZSHLF) in a positive ion flow diagram obtained by UPLC-MS/MS analysis.

UPLC-MS/MS analysis was utilized to reveal the chemical profile of ZSHLF and identify the components. Upon comparison of the MS data, 23 compounds were identified (1–23). These compounds accounted for most of the main peaks in the chromatogram and included different kinds of components, such as flavones (e.g., 12, 14, 18, and 19), phenolic acids (e.g., 11), triterpenoids (e.g., 6 and 21), and alkaloids (e.g., 4, 5, and 9) (**Figure 1** and **Table 1**).

**Table 1:** Components of Zi Shen Huo Luo Formula (ZSHLF) identified by UPLC-MS/MS analysis.

Peak No.	t_R_ (min)	Measured [M-H]^−^ (m/z)	Predicted [M-H]^−^ (m/z)	△(ppm)	Formula	Identification	Derived from
1	0.23	278.1518	278.1517	0.3	C_16_H_22_O_4_	Dibutyl phthalate	A
2	0.28	162.0681	162.0680	0.36	C_10_H_10_O_2_	Methyl cinnamate	C
3	0.35	270.2559	270.2558	0.24	C_17_H_34_O_2_	Methyl palmitate	A
4	0.85	117.0790	117.0792	−1.62	C_5_H_11_O_2_	Betaine	A
5	0.89	143.0946	143.0945	0.96	C_7_H_13_NO_2_	DL-Stachydrine	D
6	6.59	152.1201	152.1202	−0.90	C_10_H_16_O	D-(+)-Camphor	C
7	6.64	180.0423	180.0418	2.39	C_9_H_8_O_4_	Caffeic acid	B
8	6.76	290.0790	290.0787	1.05	C_15_H_14_O_6_	Catechin	F
9	6.78	335.1158	335.1148	2.85	C_20_H_17_NO_4_	Berberine	E
10	7.60	152.0473	152.0474	−0.42	C_8_H_8_O_3_	Vanillin	E
11	8.47	168.0423	168.0441	−11.02	C_8_H_8_O_4_	Vanillic acid	B
12	8.52	302.0427	302.0425	0.65	C_15_H_10_O_7_	Quercetin	A
13	8.52	464.0955	464.0953	0.31	C_21_H_20_O_12_	Quercetin-β-D-glucoside	D
14	8.53	610.1534	610.1523	0.65	C_27_H_30_O_16_	Rutin	A
15	8.65	194.0580	194.0580	−0.65	C_10_H_10_O_4_	Ferulic acid	B
16	8.80	146.0368	146.0368	−0.04	C_9_H_6_O_2_	Coumarin	C
17	8.93	220.1827	220.1826	0.70	C_15_H_24_O	(−)-Caryophyllene oxide	C
18	9.28	286.0477	286.0477	0.12	C_15_H_10_O_6_	Kaempferol	A
19	10.41	188.1049	188.1035	7.5169	C_9_H_16_O_4_	Azelaic acid	A, C
20	11.11	594.1373	594.1362	1.89	C_30_H_26_O_13_	Tiliroside	D
21	13.18	270.0528	288.0629	1.42	C_15_H_10_O_5_	Apigenin	D
22	15.19	278.1518	278.1517	0.36	C_16_H_22_O_4_	Diisobutylphthalate	A
23	23.36	284.2715	284.2709	2.33	C_18_H_36_O_2_	Stearic acid	A

A, root of Achyranthes bidentata Bl.; B, root of Scrophularia ningpoensis Hemsl.; C, bark of Cinnamomum cassia Presl.; D, aerial part of Leonurus japonicus Houtt.; E, rhizome of Coptis chinensis Franch.; and F, root cortex of Paeonia suffruticosa Andr.

### ZSHLF Combined With Perindopril Effectively Lowered the Blood Pressure and Serum ALD Levels of SHRs

**Figure 2:**
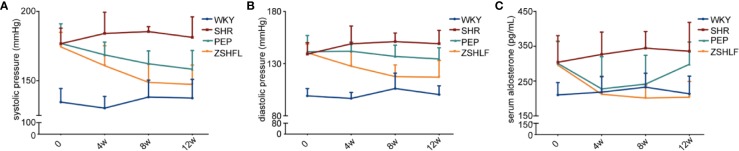
Effect of Zi Shen Huo Luo Formula (ZSHLF) on blood pressure and serum aldosterone (ALD) levels in spontaneously hypertensive rat (SHRs). **(A)** Effect of ZSHLF on systolic pressure of rats in different groups at each time point. **(B)** Effect of ZSHLF on diastolic pressure in different groups at each time point. **(C)** Effect of ZSHLF on serum ALD levels in different groups at each time point. The data represent the mean ± standard deviation, *n* = 8.

SBP and DBP were significantly higher in the SHR group than in the WKY group at each tested time point (**Supplementary Tables 2** and **3**, *P* < 0.05). Before treatment, no significant differences in SBP or DBP were found among the SHRs (*P* > 0.05). The SBP in each treatment group and the DBP in the ZSHLF group were lower than those in the SHR group at 4, 8, and 12 weeks (*P* < 0.05). Compared with that in the PEP group, the DBP in the ZSHLF group was lower at 4, 8, and 12 weeks (*P* < 0.05) (**Figures 2A, B**).

The serum ALD levels are shown in **Figure 2C**. Before and after treatment, the serum ALD levels in the SHR group were significantly higher than those in the WKY group (**Supplementary Table 4**, *P* < 0.05). Before treatment, no significant differences in serum ALD levels were found in the SHR group (*P* > 0.05). After 4 and 8 weeks of treatment, the serum ALD levels of the PEP and ZSHLF groups were significantly lower than those of the SHR group (*P* < 0.05). However, at 12 weeks, the serum ALD level of the PEP group was elevated nearly to the pretreatment value. The serum ALD level of the ZSHLF group remained low and was lower than those of the SHR and PEP groups (*P* < 0.05) (**Figure 2C**).

### Effects of ZSHLF on Left Ventricular Diastolic Function in SHRs

**Figure 3:**
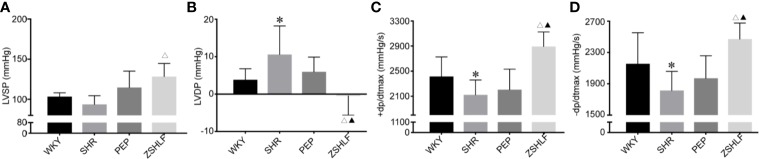
Effects of Zi Shen Huo Luo Formula (ZSHLF) on hemodynamic parameters in spontaneously hypertensive rat (SHRs). **(A)** Left ventricular hypertrophy (LVSP) of each group. **(B)** Left ventricular end diastolic pressure (LVDP) of each group. **(C)** +dp/dtmax of each group. **(D)** −dp/dtmax of each group. The data represent the mean ± standard deviation, *n*=8. *P < 0.05 vs. the WKY group; ^△^*P* < 0.05 vs. the SHR group; ^▲^*P* < 0.05 vs. the PEP group.

As shown in **Figure 3**, compared with the WKY group, the other groups exhibited decreases in ± dp/dtmax and increases in LVDP (**Supplementary Table 5**, *P* < 0.05). No significant differences in LVSP, ± dp/dtmax, or LVDP were found between the PEP and SHR groups (*P* > 0.05). However, an obvious improvements in the hemodynamic parameters were observed in the ZSHLF group compared with the SHR group (*P* < 0.05). Furthermore, LVDP was significantly decreased and ± dp/dtmax was significantly increased in the ZSHLF group compared with the PEP group (*P* < 0.05).

### ZSHLF Reduced Collagen Deposition and Inhibited the Progression of Myocardial Fibrosis in SHRs

**Figure 4:**
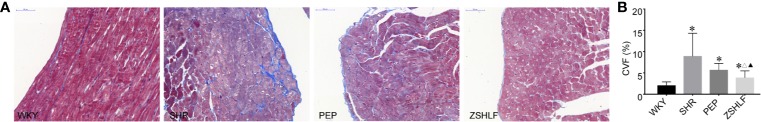
Effects of Zi Shen Huo Luo Formula (ZSHLF) on the collagen fibers of spontaneously hypertensive rat (SHRs). **(A)** Effect of ZSHLF on Masson staining in each group. **(B)** Effect of ZSHLF on the CVF in each group. The data represent the mean ± standard deviation, *n*=3. *P < 0.05 vs. the WKY group; ^△^*P* < 0.05 vs. the SHR group; ^▲^*P* < 0.05 vs. the PEP group.

As shown in **Figure 4**, Masson staining showed the presence of blue collagen fibers and red cardiomyocytes. In the WKY group, the myocardial fibers were neatly arranged, with few collagen fibers. In the SHR group, there was obvious myocardial fibrosis and significantly increased collagen composition. The cardiac CVF in the SHR group was significantly higher than that in the WKY group (**Supplementary Table 6**, *P* < 0.05). The CVF of the ZSHLF group was significantly lower than those of the SHR and PEP groups (*P* < 0.05).

### ZSHLF-Containing Serum Inhibited ALD-Induced Cardiomyocyte Hypertrophy and Cardiac Fibroblast Proliferation

**Figure 5:**
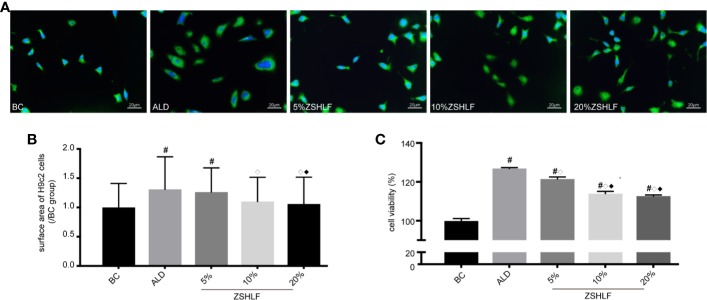
Effects of various concentrations of Zi Shen Huo Luo Formula (ZSHLF)-containing serum on the sizes of H9c2 cells and the proliferation of rat cardiac fibroblasts (RCFs). **(A)** Immunofluorescence staining of α-actinin in cardiomyocytes in different groups at different concentrations. Immunofluorescence staining was observed at 400× magnification. **(B)** Effects of ZSHLF-containing serum on cardiomyocytes surface area (10–15 cells per field, 5 fields per group; the data represent the mean ± standard deviation). **(C)** Effects of ZSHLF-containing serum on the proliferation of cardiac fibroblasts (the data represent the mean ± standard deviation, *n*=3). ^#^*P* < 0.05 vs. the BC group; ^◇^*P* < 0.05 vs. the ALD group; ^◆^*P* < 0.05 vs. the 5% ZSHLF group.

To investigate whether ZSHLF reduced cardiac hypertrophy caused by ALD *in vitro*, H9c2 cells were stimulated with ALD (10^−9^ mol/L). The cellular surface area was measured by α-actinin immunofluorescence staining, which showed that ALD-induced H9c2 cells were significantly enlarged (**Supplementary Table 7**, **Figure 5**). The cardiomyocytes surface area was significantly lower in the 10% and 20% ZSHLF groups than in the ALD group (**Figure 5B**, *P* < 0.05). There was no significant difference between the 10% and 20% ZSHLF groups (*P* > 0.05).

To investigate whether ZSHLF attenuates ALD-induced cardiac fibroblast proliferation *in vitro*, ALD was used to stimulate the proliferation of RCFs (10^−7^ mol/L). The cell viability of RCFs was assessed by CCK-8 assay after treatment. The results showed that cell viability was significantly higher in the ALD group than in the BC group (**Supplementary Table 7**, *P* < 0.05). After treatment with different concentrations of ZSHLF-containing serum, the cells in the ZSHLF serum-treated groups exhibited significantly lower viability than those in the ALD group (*P* < 0.05); furthermore, cell viability was lower in the 10% and 20% ZSHLF groups than in the 5% concentration group (*P* < 0.05). No significant differences were found between the 10% and 20% groups (*P* > 0.05).

Thus, ZSHLF-containing serum prevented ALD-induced cardiomyocyte hypertrophy and cardiac fibroblast proliferation. The 10% and 20% concentrations of medicated serum had good effects, and there was no significant difference between them. Therefore, the 10% concentration of medicated serum was selected for further study.

### ZSHLF Increased Cav-1 and MR Colocalization in ALD-Induced H9c2 Cells and RCFs

**Figure 6:**
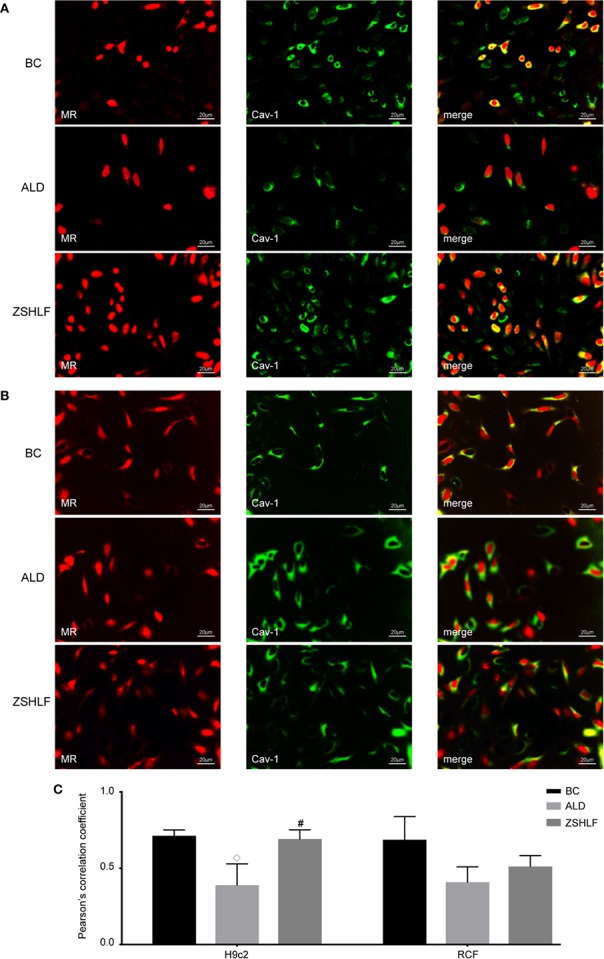
Effects of Zi Shen Huo Luo Formula (ZSHLF) on caveolin-1 (Cav-1) and mineralocorticoid receptor (MR) colocalization in aldosterone (ALD)-induced H9c2 cells and rat cardiac fibroblasts (RCFs). **(A)** Immunofluorescence labeling of Cav-1 (green) and MR (red) in H9c2 cells. **(B)** Immunofluorescence labeling of Cav-1 (green) and MR (red) in RCFs. **(C)** Effect of ZSHLF on Pearson’s r between Cav-1 and MR in H9c2 cells and RCFs. The data represent the mean ± standard deviation, *n*=3. ^#^*P* < 0.05 vs. the BC group; ^◇^*P* < 0.05 vs. the ALD group.

To investigate the molecular mechanism by which ZSHLF affected ALD-induced cardiomyocyte hypertrophy and cardiac fibroblast proliferation, changes in Cav-1 and MR colocalization in H9c2 cells and RCFs were observed. As shown in **Figure 6**, ALD decreased Cav-1 and MR colocalization in H9c2 cells and RCFs. However, compared with ALD-induced cells, H9c2 cells pretreated with ZSHLF showed marked increases in Pearson’s r for Cav-1 and MR colocalization (**Supplementary Table 8**, *P* < 0.05). Although Pearson’s r of RCFs was higher than that of the ALD-induced cells, the difference was not significant (*P* > 0.05).

### Effects of ZSHLF on the Protein Expression of EGFR and ERK in H9c2 Cells and RCFs

**Figure 7:**
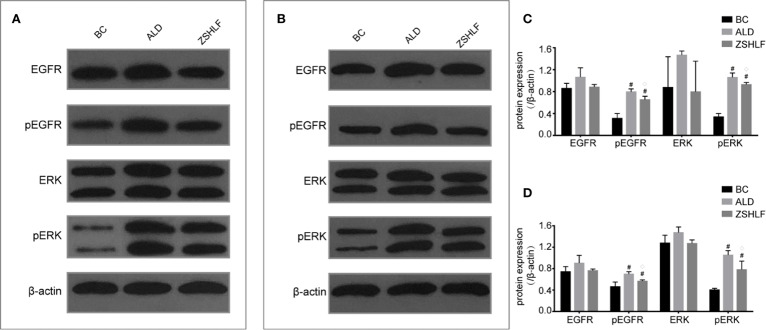
Effects of Zi Shen Huo Luo Formula (ZSHLF) on the expression of EGFR, ERK, pEGFR, and pERK in H9c2 cells and rat cardiac fibroblasts (RCFs) in different groups as assessed by western blot analysis. **(A)** The protein expression of EGFR, pEGFR, ERK, and pERK in H9c2 cells was measured by western blot analysis. **(B)** The protein expression of EGFR, pEGFR, ERK, and pERK in RCFs was measured by western blot analysis. **(C)** Relative optical density (ROD) of EGFR, pEGFR, ERK, and pERK in H9c2 cells. **(D)** ROD of EGFR, pEGFR, ERK, and pERK in RCFs. The data represent the mean ± standard deviation, *n*=3. ^#^*P* < 0.05 vs. the BC group; ^◇^*P* < 0.05 vs. the ALD group.

The western blot analysis results are shown in **Figure 7**; no significant differences in EGFR or ERK protein expression were found among the BC, ALD, and ZSHLF groups for H9c2 cells and RCFs (**Supplementary Tables 9** and **10**, *P* > 0.05). The protein expression levels of pEGFR and pERK in the two cell lines were significantly higher in the ALD and ZSHLF groups than in the BC group (*P* < 0.05). Furthermore, compared with ALD group, ZSHLF treatment inhibited pEGFR and pERK protein expression in the two cell lines (*P* < 0.05).

## Discussion

In this study, the chemical characteristics of the ZSHLF decoction were first characterized, and 23 compounds were identified by UPLC-MS/MS analysis. These findings, which can provide a basis for further screening of the active ingredients in ZSHLF. Then, the effects of ZSHLF with perindopril on ABT in the treatment of hypertensive LVH was investigated *in vivo*. All SHRs showed elevated serum ALD levels, myocardial diastolic dysfunction, and myocardial interstitial collagen fiber deposition. Perindopril reduced blood pressure and serum ALD levels, but the serum ALD levels rebounded after 12 weeks of treatment, representing the phenomenon was ABT; furthermore, perindopril could not effectively improve heart function or inhibit myocardial interstitial collagen fiber deposition. ZSHLF effectively controlled serum ALD levels, improved cardiac function and inhibited myocardial fibrosis, and the effects were better than those of perindopril alone. In vitro, ZSHLF-containing serum significantly inhibited ALD-induced cardiomyocyte hypertrophy and cardiac fibroblast proliferation. Taken together, our results suggest that ZSHLF can improve the efficacy of perindopril in the treatment of hypertensive LVH and that the effect may be related to interference with ABT and inhibition of ALD-induced pathological remodeling.

ZSHLF, a traditional Chinese compound formulated according to clinical experience, has been used clinically to treat hypertensive LVH for more than 10 years. Much evidence suggests that ZSHLF has many biological properties, including antihypertensive properties; for example, it improves hemorheology and cardiac function, and reverses LVH (Liu et al., 2013; Zhang et al., 2016; Lu et al., 2019; Mousavi et al., 2019; Yang and Lao, 2019).

We putatively identified 23 chemical compounds in ZSHLF by UPLC-MS/MS analysis. Among them, ferulic acid, caffeic acid and vanillic acid were identified to be derived from *Scrophulariae Radix*. These compounds have been reported to reduce blood pressure, improve cardiovascular structure and left ventricular function (Alam et al., 2013; Kumar et al., 2014) and exert cardioprotective effects (Agunloye et al., 2019). Berberine was found to be derived from *Coptidis Rhizoma*, which can effectively improve ventricular ejection fraction and heart function (Feng et al., 2019). Oleanolic acid, rutin, quercetin, and kaempferol were identified to have arisen from *Achyranthis Bidentatae Radix*. These compounds have been reported to have antihypertensive properties (Kaur and Muthuraman, 2016; Madlala et al., 2016; Marunaka et al., 2017) that inhibit cardiac hypertrophy and delay heart remodeling (Liao et al., 2015; Du et al., 2019). Both quercetin and rutin exhibit cardioprotective effects against isoprenaline-induced cardiac fibrosis in rats, and the mechanism may be related to inhibition of the RAS (Li et al., 2013). Stachydrine and tiliroside were identified to originate from *Leonuri Herba*. These compounds have been reported to have antihypertensive properties (Silva et al., 2013) that improve cardiac hypertrophy and myocardial fibrosis (Zhao et al., 2017). Catechin was found to come from *Moutan Cortex*, which has antihypertensive properties that inhibit angiotensin-converting enzyme (He, 2017). In summary, previous studies have confirmed that the main active constituents of ZSHLF may play roles as antihypertensive, anti-myocardial fibrosis, and cardioprotective agents and may be part of the material basis for ZSHLF function. These finding also provide guidance for our subsequent definitive constituent analysis.

Experimental and clinical evidence indicates that protracted exposure to inappropriately elevated ALD levels causes significant changes in left ventricular structure and function (Catena et al., 2015). Although the use of ACEIs and ARBs may initially decrease plasma ALD levels, chronic therapy with these drugs may lead to ABT, in which ALD levels return to or exceed the baseline levels (Struthers and MacDonald, 2004). ABT is an important phenomenon in hypertensive patients treated with ACEIs or ARBs (Soberman and Weber, 2000). Excessive secretion of ALD for long periods of time is related to pathological remodeling of myocardial tissue (Young et al., 1994; Gilbert and Brown, 2010). Therefore, ALD blockade may have added value for factors other than blood pressure in the treatment of hypertension (Hargovan and Ferro, 2014). The results of this study showed that in the PEP group, serum ALD levels tended to decrease in the early phase of treatment but increased to levels near the pretreatment values later in treatment. The serum ALD levels in the ZSHLF group remained low and were lower than those in the SHR and PEP groups. These findings suggested that the ABT phenomenon occurred during the course of perindopril treatment and that ZSHLF could effectively limit the occurrence of ABT.

It has been conclusively shown that activation of MR in the cardiovascular system promotes tissue fibrosis and has negative consequences for cardiac function following cardiac events (Young and Rickard, 2015). Classically, MR belongs to the steroid receptor superfamily, and MR effects are ascribed to ligand-receptor binding and activation of gene transcription. However, MR effects may explain the association of ALD with detrimental myocardial remodeling; ALD activation is mainly mediated by steroid receptors localized at the plasma membrane instead of by classic nuclear hormone receptor interactions (Williams, 2013). Cav-1 is an important landmark structural protein of caveolae that binds to MR and restricts its translocation and EGFR transactivation, thereby negatively regulating EGFR signaling. Inappropriate ALD levels cause direct adverse effects *via* MR actions initiated in caveolae and lead to activation of protein kinase signaling cascades (Baudrand et al., 2014). Under high-level ALD stimulation, MR is released from the cell membrane, after which it transactivates EGFR, activates the MAPK/ERK signaling pathway through c-Src, and promotes cardiomyocyte hypertrophy and fibrosis (Krug et al., 2011; Dooley et al., 2012). MR activation on the cell membrane extends the pathological features of ALD and may be an essential way to promote cardiomyocyte hypertrophy and myocardial fibrosis (Krug et al., 2011; Bienvenu et al., 2012). The results of this study showed that ALD increased the surface areas of myocardial cells, promoted the proliferation of cardiac fibroblasts, reduced Cav-1 and MR colocalization in two cell lines, and upregulated the expression of pEGFR and pERK. ALD did not affect EGFR or ERK protein expression, suggesting that ALD-induced cardiomyocyte hypertrophy and cardiac fibroblast proliferation were associated with inhibition of the binding between Cav-1 and MR, as well as promoting EGFR phosphorylation activation. ZSHLF-containing serum increased Cav-1 and MR colocalization on the cell membranes of cardiomyocytes and reduced the protein expression of pEGFR and pERK. These effects represent a possible mechanism for the inhibition of myocardial hypertrophy and fibrosis induced by ALD.

We have also summarized existing problems and future study directions. Although we analyzed the original forms of the drug-derived components of the ZSHLF decoction and medicated serum, the specific biological components or metabolites were not determined. The literature has also confirmed the antihypertensive and antifibrotic effects of the medicinal components. It is not clear which component or combination of components is responsible for reducing ABT; the issue needs further clarification. Our study found that ZSHLF can effectively control ALD levels, but aldosterone release is regulated in various ways, and whether other mechanisms participate in the observed effects should be further studied. In addition, this study involved investigation and validation only in experimental studies; observation of and research on related clinical patients have not yet been performed. We plan to conduct further research on potential applications in patients.

## Conclusions

In summary, the current findings suggest that ZSHLF could interfere with ABT and suppress ALD-induced cardiomyocyte hypertrophy and cardiac fibroblast proliferation by affecting MR and Cav-1 colocalization and the downstream EGFR signaling pathway. These effects may represent one of the mechanisms by which ZSHLF improves the efficacy of ACEIs in reversing hypertensive LVH.

## Data Availability Statement

All datasets generated for this study are included in the article/**Supplementary Material**.

## Ethics Statement

The animal study was reviewed and approved by Welfare Ethics Committee of Experimental Animals of Shandong University of Traditional Chinese Medicine.

## Author Contributions

ZW and HY conceived and designed the experiments. XS wrote the manuscript. YZ and QC participated in the animal experiments. HY and HW participated in the cell experiments. SW, YW, and HZ helped perform the analyses with constructive discussions. ST carried out the statistical analyses. All authors have read and approved the manuscript.

## Funding

This work was supported by the National Natural Science Foundation of China (No. 81503539).

## Conflict of Interest

The authors declare that the research was conducted in the absence of any commercial or financial relationships that could be construed as a potential conflict of interest.
