# Supplementary material for: Zi Shen Huo Luo Formula Enhances the Therapeutic Effects of Angiotensin-Converting Enzyme Inhibitors on Hypertensive Left Ventricular Hypertrophy by Interfering With Aldosterone Breakthrough and Affecting Caveolin-1/Mineralocorticoid Receptor Colocalization and Downstream Extracellular Signal-Regulated Kinase Signaling
Source: Front Pharmacol. 2020 Apr 3;11:383. doi: 10.3389/fphar.2020.00383 (PMC7147343; doi:10.3389/fphar.2020.00383)
Supplement: Supplementary file 1 [file DataSheet_1.docx]

Supplementary Material

**Supplementary Table 1 |** Detailed information of the crude drugs composed in ZSHLF

| Herbal names | botanical names | Chinese name | Place of Origin | Voucher Specimens Reserve |
| --- | --- | --- | --- | --- |
| Scrophulariae Radix | Scrophularia ningpoensis Hemsl. | 玄参 | Zhejiang, China | Affiliated Hospital of Shandong University of Traditional Chinese Medicine |
| Achyranthis Bidentatae Radix | Achyranthes bidentata Blume. | 怀牛膝 | Henan, China | Affiliated Hospital of Shandong University of Traditional Chinese Medicine |
| Coptidis Rhizoma | Coptis chinensis Franch. | 黄连 | Sichuan, China | Affiliated Hospital of Shandong University of Traditional Chinese Medicine |
| Moutan Cortex | Paeonia suffruticosa Andr. | 牡丹皮 | Anhui, China | Affiliated Hospital of Shandong University of Traditional Chinese Medicine |
| Leonuri Herba | Leonurus japonicus Houtt. | 益母草 | Guangdong, China | Affiliated Hospital of Shandong University of Traditional Chinese Medicine |
| Cinnamomi Cortex | Cinnamomum cassia Presl | 肉桂 | Guangxi, China | Affiliated Hospital of Shandong University of Traditional Chinese Medicine |

**Supplementary Table 2 |** Effect of ZSHLF on SBP in rats (mmHg，)

| group | 0 | 4w | 8w | 12w |
| --- | --- | --- | --- | --- |
| WKY | 134.63±9.85^△^ | 130.29±8.43^△^ | 138.29±12.17^△^ | 137.50±13.47^△^ |
| SHR | 176.71±11.14 | 184.13±15.28 | 185.42±3.54 | 181.29±14.75 |
| PEP | 176.88±14.15 | 168.69±9.20^△^ | 162.23±9.23^△^ | 158.33±13.52^△^ |
| ZSHLF | 174.38±10.53 | 160.88±14.35^△^ | 148.83±13.43^△▲^ | 147.38±13.94^△▲^ |

^△^ *P*<0.05 vs. SHR group; ^▲^ *P*<0.05 vs. PEP group.

**Supplementary Table 3 |** Effect of ZSHLF on DBP in rats (mmHg，)

| group | 0 | 4w | 8w | 12w |
| --- | --- | --- | --- | --- |
| WKY | 99.04±7.11^△^ | 96.63±5.73^△^ | 106.12±14.69^△^ | 100.33±8.40^△^ |
| SHR | 139.04±10.93 | 148.96±16.95 | 151.00±8.32 | 149.04±12.79 |
| PEP | 141.33±15.55 | 141.83±8.62 | 136.81±10.76^△^ | 134.42±10.73^△^ |
| ZSHLF | 140.33±8.29 | 127.54±14.08^△^ | 117.54±11.21^△▲^ | 116.88±16.13^△▲^ |

^△^ *P*<0.05 vs. SHR group; ^▲^ *P*<0.05 vs. PEP group.

**Supplementary Table 4 |** Effect of ZSHLF on serum aldosterone levels in rats(pg/ml，)

|  | 0 | 4w | 8w | 12w |
| --- | --- | --- | --- | --- |
| WKY | 210.36±35.39^△^ | 218.49±44.04^△^ | 232.51±39.92^△^ | 213.55±50.74^△^ |
| SHR | 304.11±76.20 | 326.62±64.24 | 344.56±47.75 | 335.51±82.80 |
| PEP | 300.60±62.83 | 227.57±92.44^△^ | 241.33±82.64^△^ | 298.19±63.95 |
| ZSHLF | 295.77±69.34 | 212.28±51.89^△^ | 201.26±37.63^△^ | 203.79±45.13^△▲^ |

^△^ *P*<0.05 vs. SHR group; ^▲^ *P*<0.05 vs. PEP group.

**Supplementary Table 5 |** Effects of ZSHLF on hemodynamic parameters in rats ()

|  | LVSP（mmHg） | LVDP  （mmHg） | +dp/dtmax  (mmHg/s) | －dp/dtmax  (mmHg/s) |
| --- | --- | --- | --- | --- |
| WKY | 103.35±4.79 | 3.82±2.97^△^ | 2415.22±310.86^△^ | -2155.24±396.66^△^ |
| SHR | 93.71±10.81 | 10.60±7.62 | 2123.26±234.94 | -1814.60±244.41 |
| PEP | 114.76±20.43 | 5.97±3.93 | 2204.37±327.91 | -1970.85±287.60 |
| ZSHLF | 128.35±16.30^△^ | -0.47±5.17^△▲^ | 2892.86±232.24^△▲^ | -2471.53±204.90^△▲^ |

^△^ *P*<0.05 vs. SHR group; ^▲^ *P*<0.05 vs. PEP group.

**Supplementary Table 6 |** Effects of ZSHLF on collagen volume fraction in rats (%, )

|  | CVF（%） |
| --- | --- |
| WKY | 2.08±0.81^△^ |
| SHR | 8.97±5.33 |
| PEP | 5.72±1.52 |
| ZSHLF | 3.90±1.58^△▲^ |

^△^ *P*<0.05 vs. SHR group; ^▲^ *P*<0.05 vs. PEP group.

**Supplementary Table 7 |** Effects of various concentrations ZSHLF-containing serum on cell size of H9c2 and proliferation of RCF ()

| group | Cell surface area（/BC） | OD（CCK-8） |
| --- | --- | --- |
| BC | 1.000±0.412^△^ | 0.441±0.006^△^ |
| ALD | 1.310±0.556 | 0.560±0.002 |
| 5% ZSHLF | 1.264±0.413 | 0.536±0.005^△^ |
| 10% ZSHLF | 1.102±0.415^△^ | 0.503±0.005^△▲^ |
| 20% ZSHLF | 1.061±0.457^△▲^ | 0.497±0.003^△▲^ |

^△^ *P*<0.05 vs. ALD group; ^▲^ *P*<0.05 vs. 5% ZSHLF group.

**Supplementary Table 8 |** Effects of ZSHLF-containing serum on Colocalization Pearson’s Correlation Coefficient in H9c2 and RCF ()

| group | H9c2 | RCF |
| --- | --- | --- |
| BC | 0.713±0.038^△^ | 0.687±0.152^△^ |
| ALD | 0.390±0.139 | 0.409±0.100 |
| ZSHLF | 0.692±0.060^△^ | 0.512±0.071 |

^△^ *P*<0.05 vs. ALD group.

**Supplementary Table 9 |** Effects of ZSHLF-containing serum on EGFR and ERK protein expression in H9c2 cell ()

| group | EGFR | pEGFR | ERK | pERK |
| --- | --- | --- | --- | --- |
| BC | 0.868±0.084 | 0.321±0.079^△^ | 0.886±0.550 | 0.346±0.055^△^ |
| ALD | 1.071±0.163 | 0.804±0.044 | 1.475±0.067 | 1.068±0.071 |
| ZSHLF | 0.892±0.038 | 0.659±0.056^△^ | 0.804±0.552 | 0.936±0.030^△^ |

^△^ *P*<0.05 vs. ALD group.

**Supplementary Table 10 |** Effects of ZSHLF-containing serum on EGFR and ERK protein expression in RCF cell ()

| group | EGFR | pEGFR | ERK | pERK |
| --- | --- | --- | --- | --- |
| BC | 0.751±0.086 | 0.472±0.077^△^ | 1.288±0.136 | 0.413±0.019 ^△^ |
| ALD | 0.912±0.136 | 0.708±0.036 | 1.482±0.096 | 1.060±0.077 |
| ZSHLF | 0.772±0.021 | 0.575±0.017^△^ | 1.280±0.055 | 0.790±0.149^△^ |

^△^ *P*<0.05 vs. ALD group.
